# Orchestrating brain fluid dynamics: circadian regulation of CSF, glymphatic exchange, and waste clearance

**DOI:** 10.3389/fcell.2026.1891553

**Published:** 2026-09-03

**Authors:** Clara Ciampi, Stefano Govoni, Stefania Preda, Lorenzo Di Cesare Mannelli, Marco Racchi, Alma Martelli, Cristina Lanni

**Affiliations:** 1 Department of Neuroscience, Psychology, Drug Research and Child Health-NEUROFARBA, Pharmacology and Toxicology Section, University of Florence, Florence, Italy; 2 Department of Drug Sciences, University of Pavia, Pavia, Italy; 3 Department of Pharmacy, University of Pisa, Pisa, Italy

**Keywords:** astrocyte, choroid plexus, clock genes, glymphatic system, neurovascular unit

## Abstract

The brain relies on cerebrospinal fluid (CSF) and the glymphatic system to maintain its delicate internal environment. We explored through a literature search the complex interplay and circadian regulation of CSF production and glymphatic system exchanges attempting to delineate an integrated and innovative view of their activities. We focused on how biological clocks, present at various levels of the finely tuned CSF/interstitial fluid/glymphatic exchange chain, orchestrate these activities. A key aspect is exploring their relationship with the neurovascular unit (NVU), which acts as a crucial regulator of fluidic control and exchanges at the neuron/astrocyte/endothelium interface. While the daily cycling activity of the glymphatic waste system is supported by increasing experimental evidence in animal models, the precise coordination and the primary location of the underlying biological clocks remain subjects of ongoing discussion. We provided a comprehensive discussion addressing these unanswered questions regarding the synchronization and primary regulatory mechanisms governing brain fluid dynamics. Understanding the circadian orchestration of CSF and glymphatic system activities, and their intricate connections with the NVU, is paramount for comprehending brain homeostasis and disease. Further research into the precise localization and function of these biological clocks is crucial for advancing our knowledge of brain fluid dynamics.

## 1 Introduction

The brain, similar to other organs, possesses a sophisticated perfusion system and precise regulation of the fluids permeating its parenchyma. Within the brain, various interconnected compartments and interstitial spaces facilitate the delivery of nutrients, the circulation of regulatory factors, and the removal of waste. This transport is increasingly understood as a multimodal process, where solute movement is likely governed by concurrent mechanisms. This includes convective (advective) flow within perivascular spaces, which facilitates directional transport (Iliff et al., 2012; Nedergaard, 2013), and diffusional transport driven by concentration gradients, which contributes significantly to the distribution of molecules within the dense interstitial matrix (Syková and Nicholson, 2008; Asgari et al., 2016).

Blood and the cerebrospinal fluid (CSF) are the fluids circulating in the brain. Blood, mostly providing energy and the delivery of peripheral low molecular weight signalling substances, is barriered at all levels of the vascular tree, to avoid the entry within the brain parenchyma of large molecular species (either originating from the body or coming from the diet/environment) eventually perturbing neuronal activity. Tight junctions at endothelial level and transport systems subserve this function. The other ventriculo-subarachnoid compartmental network is provided by the subarachnoid space and connected to the ventricular system, ending with the spinal central canal. The CSF is mostly produced at the level of the choroid plexuses (CP), even if an extra-choroidal production may also contribute (Orešković and Klarica, 2010). The CSF is also barriered toward blood and brain and may subserve exchanges and delivery of signalling molecules as well as waste products removal. The flow driven by various signals and active/passive mechanical drivers moves the CSF toward the subarachnoid spaces and through the brain parenchyma, where it maintains relationships and exchanges with the interstitial fluid (ISF) and the glymphatic system (GS).

A perivascular fluid conduit system is provided by the GS with periarterial and perivenous spaces that may collect waste material and have active exchanges with the parenchymal extracellular fluid.

Glymphatic clearance facilitates the movement of interstitial fluid toward the subarachnoid space, from where it is subsequently drained via meningeal lymphatic vessels to the cervical lymph nodes.

The GS is considered a crucial element in the rhythmic daily activities of neuronal activity waste removal from the brain (Xie et al., 2013; Hablitz et al., 2020).

The present review deals with the activity and relationships between the CSF production and GS exchanges, and, in particular, with their circadian regulation. The term circadian refers to the endogenous, self-sustained biological rhythms that oscillate within a period of approximately 24 h. These rhythms are orchestrated by a hierarchical system comprising a master pacemaker in the suprachiasmatic nucleus (SCN) of the hypothalamus and peripheral oscillators located in almost every tissue, including the brain’s glial cells. At the molecular level, these circadian oscillations are driven by an evolutionary conserved transcriptional-translational feedback loop (TTFL) (Hastings et al., 2018; Fagiani et al., 2022). Briefly, the core molecular clock consists of the transcription factors CLOCK and BMAL1, which heterodimerize to activate the expression of the Period (*Per1*–3, encoding the proteins PER1–PER3) and Cryptochrome (*Cry1*–2, encoding CRY1–CRY2) genes. As PER and CRY proteins accumulate in the cytoplasm, they form complexes that translocate back into the nucleus to inhibit the activity of CLOCK:BMAL1, thereby suppressing their own transcription. This self-sustaining cycle takes approximately 24 h to complete and is further refined by secondary loops involving nuclear receptors such as REV-ERBα, ensuring the rhythmic regulation of downstream clock-controlled genes, governing cellular metabolism and perivascular flow.

The primary objective of this review is to illustrate how biological clocks, operating at different physiological scales, orchestrate the production, composition, and exchange of fluid between the CSF and interstitial fluid (ISF) across ventricular, perivascular, and interstitial compartments. We examined these mechanisms within the theoretical framework of the GS and its structural integration with the neurovascular unit (NVU), the fundamental regulator of fluid dynamics and solute clearance at the neuron-astrocyte-endothelium interface.

Importantly, while the circadian rhythmicity of individual systems, such as the glymphatic network, is increasingly recognized, direct evidence regarding the integrated circadian coordination across the choroid plexus, the NVU, and the GS remains limited. To address this gap, this review synthesizes current data to propose a cohesive, multi-system perspective, connecting these distinct anatomical segments and highlighting a critical, yet largely unexplored, dimension of brain fluid homeostasis.

## 2 Methods

A literature search was carried out for peer-reviewed research published up until June 2026 in the PubMed and Scopus databases to guarantee transparency and rigor. “Glymphatic system”, “cerebrospinal fluid”, “choroid plexus”, “CSF composition”, “aquaporin 4”, “aquaporin 1”, “transporters”, “bulk flow”, “diffusion”, “convection”, “fluid drainage”, “circadian rhythm”, “clock genes” (e.g., *Bmal1*, Clock, *Per2*, *Cry1*), “neurovascular unit”, “blood-brain barrier” and “perivascular” were among the terms that were combined with text keywords in the search method. Clinical studies, basic animal models, and original research publications in *in vitro* and *in vivo* models were combined in this narrative review. Studies assessing the connections between biological clocks and cerebral fluid compartment dynamics were the main focus of the inclusion criteria. Papers describing the normal anatomical and cellular structure and the basic physiological functions were also selected when appropriate. Excluded papers were descriptive studies related to the architecture of sleep without evaluating fluid mechanics or molecular oscillators.

## 3 Choroid plexus

### 3.1 Generating CSF and sustaining its flux through the brain: the role of choroid plexuses

The CSF is mainly produced at the level of CP in the lateral ventricles, where the blood-cerebrospinal fluid barrier (BCSFB) operates. Furthermore, the net secretion of water across the CP constitutes the bulk flow of CSF, which, however, may also be regulated by different processes at the various levels participating to this complex brain fluidic control (Takagishi et al., 2024). In this way, and due to the large surface area of BCSFB, the flux sustains the circulation of compounds useful for brain functioning and the removal of waste products in the passage through the ventricular brain system and brain parenchyma toward its efflux sites (Johanson, 2018). The CP production of CSF is supported by a highly folded basolateral membrane (Liddelow, 2015), a luminal membrane covered by short microvilli, that can increase its surface area by up to 15 times, a high density of mitochondria, connections made by tight junctions pointing toward the luminal aspect, and expression of multiple membrane transport mechanisms (Oernbo et al., 2022).

About 500 mL of CSF are secreted into the brain each day by the vasculature in an adult individual (Margetis et al., 2025). The ventricular system is the passageway for the CSF.

Before the fluid enters the spinal canal or the subarachnoid space, it is driven by the hydrostatic pressure that results from its continuous secretion within the ventricles, and by the directed beating of the cilia of the ependymal cells lining the ventricular walls that promote the laminar CSF flow within ventricles and the aqueduct (Takagishi et al., 2024). CSF flow is also determined by the respiratory cycle and the arterial pulsations (Gutiérrez-Montes et al., 2022; Ozturk et al., 2023; Vijayakrishnan Nair et al., 2022; Liu et al., 2022), and it can be modulated by body posture (Alperin et al., 2005; Muccio et al., 2021), exercise (Miyazaki et al., 2024; Wright et al., 2025), and the state of brain activity (Miyakoshi et al., 2023), including the sleep/wake cycle (Uji et al., 2025) and anesthesia (Stanton et al., 2021; Liu et al., 2020; Hablitz et al., 2019). Upon reaching the subarachnoid space, CSF undergoes partial absorption into the venous blood through arachnoid granulations (McKnight et al., 2020). Additionally, CSF through the periarterial spaces surrounding penetrating arterioles reaches the brain parenchyma, driven by the pulsation of arterial walls, thus supporting the transport of substances and the exchange between CSF and the ISF, then exits through perivenous spaces (surrounding venules) towards the arachnoid granulations and the arachnoid cuff exit to maintain fluid balance and final elimination of waste products (Kipnis, 2024). CSF also drains to extracranial lymph nodes via cranial nerve sheaths, particularly those of the spine, olfactory, and optic nerves (Proulx, 2021).

The CSF shares a similar composition with the brain ISF due to the continuous exchange of substances, whereas it shows a different composition compared to blood (Davson and Segal, 1996; Hladky and Barrand, 2014). The similar composition of CSF and ISF is particularly referred to ionic composition (Na^+^, K^+^, Ca^2+^, Mg^2+^, HCO_3-_, Cl^−^) (Hladky and Barrand, 2014) sampling the ISF in the cerebral cortex and the CSF in the cisterna magna as summarized in (Hansen, 1985). In particular, due to physical barriers limiting the free substance exchange between blood and CSF, the latter has an up to more than two orders of magnitude lower content of proteins and cholesterol (Borràs et al., 2025). CSF composition includes the electrolytes potassium (K^+^), calcium (Ca^2+^), sodium (Na^+^), chloride (Cl^−^), bicarbonate (HCO_3−_) and magnesium (Mg^2+^), proteins, sugars, amino acids, creatinine, uric acid, urea, cholesterol, lactic acid, and inorganic phosphate (Lyckenvik et al., 2025).

The ion composition of CSF is regulated by active transport mechanisms, including transporters, cotransporters and ion channels present on epithelial cells of the CP, that ensure the unidirectional passage of ions from basolateral membrane to cytoplasm, then across apical membrane into ventricles (MacAulay et al., 2022). Hence, CSF production is regulated by a balanced, rhythmic, and interconnected sequence of events essential for maintaining osmolarity and ensuring its continuity. In particular, the secretion of Na^+^, Cl^−^, and HCO_3_^−^ and the absorption of K^+^ are crucial for maintaining the osmotic gradient necessary for water secretion, which is the major component (99%) of CSF (Czarniak et al., 2023; Lolansen et al., 2025).

**FIGURE 1:**
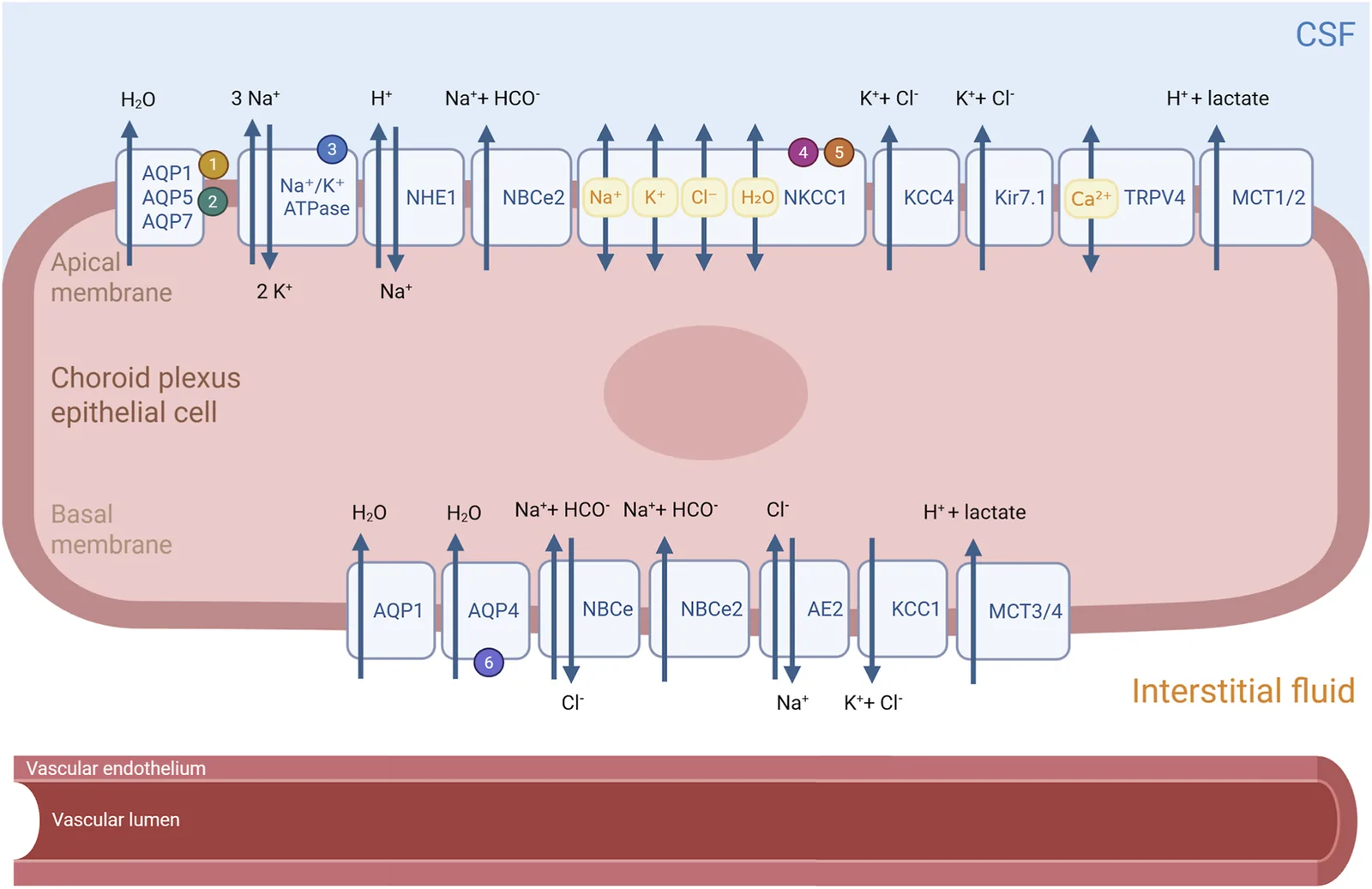
Choroid plexus transporters, cotransporters and ion channels/exchangers present on the basal and apical membrane of the epithelial choroid plexus cell and their regulation. Numbers in figure indicate: 1) AQP1: No circadian rhythm; 2) AQP5: Its activity has been associated with brain edema and CSF homeostasis; 3) Na^+^/K^+^ ATPase: Sensitive to carbachol, ouabain; 4) NKCC1: Regulated by TRPV4, WNK1; 5) NKCC1: elevated during the dark phase; 6) AQP4: May compensate for reduced CSF secretion.

Water movements do not occur passively, rather, the water flow is a regulated passage driven by specifically expressed water channels, as well as by the control of the paracellular flow by the regulation of tight junctions’ strength through adhesion proteins expression. Several aquaporin (AQP)-water channels are present in CP (Figure 1). In particular, aquaporin-1 (AQP1), localized in the lumen-facing membrane of CP ependymal cells is one of the best investigated for its role in CSF production. The genetic deletion of AQP1 in mice has been found to decrease by 20%–25% the CSF secretion rate (Oshio et al., 2005). AQP1 in the CP has been demonstrated to be functionally interconnected with aquaporin-4 (AQP4) acting at the nonchoroidal level to finely balance and regulate CSF secretion, as also indicated in human brain (Bihlmaier et al., 2023). While AQP1 facilitates the passive transport of water through the apical membrane of CP epithelial cells, AQP4, by residing in ependymal cells and astrocytes, provides the major nonchoroidal contribution to CSF formation, as shown by Trillo-Contreras and colleagues (Trillo-Contreras et al., 2019) working on AQP1^−/−^ and AQP4^−/−^ mice. Although these results emphasize the crucial role that astrocytic and ependymal AQP4 play in fluid movement, other possible parallel sources or transport mechanisms that could be responsible for the formation of nonchoroidal CSF, have not been assessed. Within the frame of the present review, such sources include, for example, the subarachnoid arterioles actively secreting CSF-like fluid directly in the subarachnoid space, and the ISF of the brain parenchyma (Hladky and Barrand, 2014).

Hence, a more quantitative analysis is needed to determine the precise fractional contribution of AQP4 in relation to other plausible extrachoroidal fluid routes.

While AQPs facilitate passive water transport driven by osmotic gradients, the CP epithelium has been shown to transport water against such gradients through various mechanisms, as mentioned above and as summarized in Figure 1, based on cotransporters, energy dependent pumps and adhesion molecules controlling cell to cell tight junctions. This capacity to transport water against an osmotic gradient aligns more closely with the active transport properties (MacAulay and Toft-Bertelsen, 2023; Steffensen et al., 2018; Jensen et al., 2025). Notably, all these elements can be modulated by drugs, such as diuretics and digitalis glycosides (Uldall et al., 2017; Biringer, 2025).

The production of CSF is also regulated by various nervous mechanisms, including sympathetic, cholinergic, serotonergic, and peptidergic influences. All these systems in normal physiological conditions have an oscillating daily activity driven by both internal clocks (such as the sympathetic/vagal activity balance) as well as by peripheral signals which may function as external synchronizing events (for example, food intake) or as emergency signals disrupting the normal rhythm (as consequence of a strong stress event) (Vizcarra et al., 2024; Myung et al., 2018; Yamaguchi et al., 2020; Quintela et al., 2018a; Quintela et al., 2021; Quintela et al., 2018b; Quintela et al., 2015; Fame et al., 2023).

Figure 1 summarizes the various transporters, cotransporters and ion channels/exchangers present on the basal and apical membrane of the choroid plexus epithelial cells. The complexity of the organization of the system and of the signals regulating it is so far only partially explored and understood.

### 3.2 Rhythmic fine-tuning CSF: the relevance of the circadian clockwork

Literature data on a circadian regulation at CP level are still scarce and fragmented.

CP operates on a circadian timescale, synchronized with the 24-h day-night cycle (Myung et al., 2018), under control of the (SCN) (Sládek et al., 2024).

Surgical lesion of SCN in control mice impacts on CP leading to a significant reduction in the rhythmic expression of specific genes, such as *Kcne2*, which encodes a regulatory subunit of potassium channels, and *Slc22a8* gene, encoding a protein involved in the sodium-independent transport (Sládek et al., 2024).

Astrocytes in the SCN show peak oscillations of Ca^2+^ during the night in clear opposition to the peak of the SCN neurons during the day (Brancaccio et al., 2019). Furthermore, SCN astrocytes show oscillations in the release of glutamate in correspondence with the Ca^2+^ peak, taken up by neurons in a rhythmic manner through the time-of-day dependent activity of excitatory amino acid transporter 3 (EAAT3) (Brancaccio et al., 2017). Moreover, astrocytic modulation of GABA signalling under the control of *Bmal1* has been described (Brancaccio et al., 2019). Therefore, the SCN shows both clock neurons active during the day and astrocytes active at night (Costa and Montagnese, 2021; Albrecht and Ripperger, 2018).

SCN links with CP also through CSF that transports diffusible signals which are biomarkers of circadian rhythmicity of various functions, not limited to CSF production. These signals include, for example, the arginine vasopressin (AVP) (Tousson and Meissl, 2004) and the vasoactive intestinal polypeptide (VIP) (Nilsson et al., 1992), melatonin (Tan et al., 2016), serotonin (Yan et al., 1993), transthyretin (Fame et al., 2023), as also summarized in (Vizcarra et al., 2024). Furthermore, endogenous glucocorticoids may act as circadian signal present in CSF. Indeed dexamethasone (DEX) impacts on the circadian clock in CP, enhancing the amplitude of circadian oscillation and restoring the phase of the CP clock in both *in vivo* and *in vitro* models. Additionally, DEX activates Period circadian protein homolog 1 (*Per1*) and Period Circadian Regulator 2 (*Per2*) turnover, highlighting the role of glucocorticoids in reinforcing and synchronizing the CP circadian rhythm, which is crucial for regulating CSF production according to the time of day (Liška et al., 2021).

CP has been further identified as an extra-pineal source of melatonin in the brain (Quintela et al., 2018a). Melatonin synthesized by the CP showed a paracrine effect on the BCSFB, influencing the expression of tight junction proteins, such as claudin-2, and revealing a self-regulating rhythmic permeability mechanism of the BCSFB (Quintela et al., 2021).

Notably, SCN receives direct entrainment signals from the external light-dark cycle and is thought to propagate information to the CP, and to the pineal gland (PG) as discussed by Yamaguchi et al. in transgenic male rats based on Wistar background strain (Yamaguchi et al., 2020). The authors propose a model of signal trafficking where the SCN transmits information possibly via noradrenergic fibers to PG and to CP, whereas it is hypothesized that the PG influences the period of SCN and of CP by melatonin secretion via CSF (Yamaguchi et al., 2020). It should be noted, however, that while Yamaguchi et al., (2020) observed rhythmic clock gene expression, the specific involvement of noradrenergic fibers and CSF-mediated melatonin transport in this context remains an area for further experimental validation.

In particular, the quoted paper investigated the synchronization among three main players, CP, PG and SCN in explant conditions and *in vivo*. In explant conditions, where tissues are decoupled from systemic entrainment, they observed distinct intrinsic free-running periods. The SCN exhibited a period of about 25 h, consistent with established observations of the rat master clock’s intrinsic frequency *in vitro* (Welsh et al., 1995). Interestingly, the CP (both CP-4V and CP-LV) presented a significantly shorter sustained period of roughly 21 h, that persisted for several days in isolation, suggesting the presence of an internal oscillator. Despite these divergent intrinsic periodicities, the PG maintained a 24-h rhythm in both *in vivo* and explant conditions, leading the authors to suggest it may act as a stabilizing frequency-coupler within this circuit. This indicates that while these components possess autonomous oscillatory capabilities, they are hierarchically synchronized *in vivo* to a 24-h period, potentially through the PG’s integration of SCN-derived signals. When analyzing the circadian period in these organs following explant compared to the period assessed in *in vivo* conditions, explants of the fourth (CP-4V) and lateral ventricle (CP-LV) presented a period of 21 h, and the explant of SCN showed a period of 25-h compared to 24-h period in *in vivo* conditions (Yamaguchi et al., 2020). These results showed that, in these experimental conditions, assuming that the transgene does not influence the system, both CP and SCN aligned with the PG, which maintained its circadian period of 24-h both in explants and *in vivo* conditions. Accordingly, the circadian rhythm of the system is synchronized on the 24hrs period in spite of intrinsic oscillators of the various components having a different phase and period, opening the question concerning their regulation in the case of derailment of the oscillator of one of the components or of the synchronizing external signal.

As a consequence, CSF production is under circadian regulation and CSF distribution between brain areas also varies across the 24-h day (Hablitz et al., 2020). Steffensen et al., studied the fluctuation of CSF production peaks during the dark phase in humans and rodents (Steffensen et al., 2023). This nocturnal surge is driven by upregulated choroid plexus (CP) transporters: the Na^+^-driven Cl^−^/HCO_3-_ exchanger (NCBE) (+11%) and the sodium bicarbonate cotransporter 3 (NBCn1) (+21%) at Zeiteberg Time (ZT) 20 compared to ZT 8. Additionally, increased nighttime secretion involves elevated Na–K–Cl cotransporter 1 (NKCC1) activity, modulated by an increase in transient receptor potential vanilloid 4 (TRPV4) and lysine deficient protein kinase 1 (WNK1) (Steffensen et al., 2023).

The altered ionic transport gives origin to fluid exchanges between compartments that need permeability changes. Given that AQP1 does not appear to be directly under circadian control (Yamaguchi et al., 2020), or its role in circadian regulation remains unclear, there must be other mechanisms that facilitate the increased permeability of water across the barrier during the night. On the other hand, recently published data in Wistar rats (Yamaguchi et al., 2026) raise the possibility that AQP1 undergoes diurnal variations in the rat choroid plexus for cerebrospinal fluid secretion, together with claudin-2 likely participating in the changes in CSF secretion, but not aligned with the night CSF production peaks observed both in man and rodents.

In contrast, AQP4 localization and functionality have been found to be regulated by circadian rhythms (Hablitz et al., 2020), although this happens mostly at extrachoroidal level.

The increased water secretion from the blood side into the CSF during the dark phase appears to be related to the activation of claudin-2 expression influenced by melatonin. Although there is no evidence that claudin-2 expression is caused by CP-produced melatonin, in ovine it has been shown that slow-release melatonin implants upregulate claudin-2 in choroid plexus, leading to increased permeability of the blood-cerebrospinal fluid barrier, in turn increasing the flow of water and other substances between the blood and CSF (Szczepkowska et al., 2019).

To fully appreciate these rhythmic modifications in barrier function, it is essential to underscore the distinct molecular architecture of paracellular transport within the choroid plexus epithelium (CPE) compared to the vascular endothelium of the BBB. While the brain capillary endothelial cells establishing the BBB rely heavily on a highly restrictive tight junction meshwork characterized by prominent expressions of occludin, ZO-1, and claudin-5 to seal the paracellular space, the epithelial BCSFB exhibits structural and cell-specific variations.

Notably, claudin-5, which represents a fundamental sealing component of the endothelial BBB, is entirely absent from the CPE of mice, where paracellular sealing is instead dominated by other isoforms like claudin-1 and claudin-11 (Steinemann et al., 2016). In the CPE, the paracellular pathway behaves as a dynamic pore system. An example is provided by claudin-2, which is enriched in the apical membranes of choroid plexus epithelial cells and forms cation- and water-permeable paracellular channels. As previously mentioned, claudin-2 can be upregulated by nocturnal melatonin surges, directly facilitating the paracellular transit of water to support the dark-phase peak of CSF secretion. This molecular heterogeneity underscores that while the endothelial BBB is optimized for rigid restriction, the epithelial BCSFB is uniquely specialized to accommodate regulated fluid and ion exchange, partially via paracellular channels, under circadian control.

In summary, observations showing increased CSF production during the dark phase correlated with changes in the expression of transporters and exchangers, suggesting that circadian regulation is influenced by signals potentially mediated by clock genes. Specifically, *Per2* might play crucial roles in coordinating transporter activity modulating the permeability of BCSFB. *Per2* could be implicated in the regulation of genes responsible for producing proteins like Claudin-2, which affect the permeability of the BCSFB, and consequently, CSF secretion (Quintela et al., 2021).

### 3.3 Circadian CSF composition changes and hierarchical clock organization

Findings indicate that the composition of CSF not only includes biomarkers influenced by circadian rhythms but also may have significant functions in transmitting daily rhythmic information to brain tissues (Quintela et al., 2021). The metabolite composition of rat CSF may vary both in light phase (ZT 8 h) and dark phase (ZT 20 h) (Edelbo et al., 2023). In particular, melatonin, urocanic acid, and 5-methoxytryptophan (a melatonin and serotonin precursor) increase in the dark phase, whereas lysine, cytosine, arginine, and 5-methylcytosine, among others, increase in the light phase (Edelbo et al., 2023). A fluctuation in CSF composition is related to a transcriptomic profile that varies during the day. RNA-seq on CP excised from rats in the peak light phase (ZT 8 h) and in the peak dark phase (ZT 20 h) demonstrated significant changes in the dark/light transcriptomic and metabolomic (Edelbo et al., 2023) profiles.

The SCN acts as the body’s master circadian pacemaker, characterized by endogenous, self-perpetuating oscillations that persist even in the absence of external cues. While these rhythms are generated internally at the cellular level, they are entrained to the 24-h solar day via external stimuli, such as light input from the retino-hypothalamic tract, food intake, and motor activity. The pivotal role of the SCN has been demonstrated through lesion studies spanning several decades, which show that its destruction leads to a complete loss of behavioral and physiological rhythmicity (summarized in (Welsh et al., 2010)). Also the analysis by Hastings et al. (2018) underscores that the SCN is a uniquely precise and robust clock. More recently, research has highlighted how SCN-driven signals maintain synchrony in peripheral tissues; for instance, SCN lesions lead to the dysregulation of circadian genes within the choroid plexus, specifically *Nr1d1* (Rev-Erbα), *Ciart* (*Chrono*), *Arntl* (*Bmal1*), and *Dbp* (Sládek et al., 2024).

**FIGURE 2:**
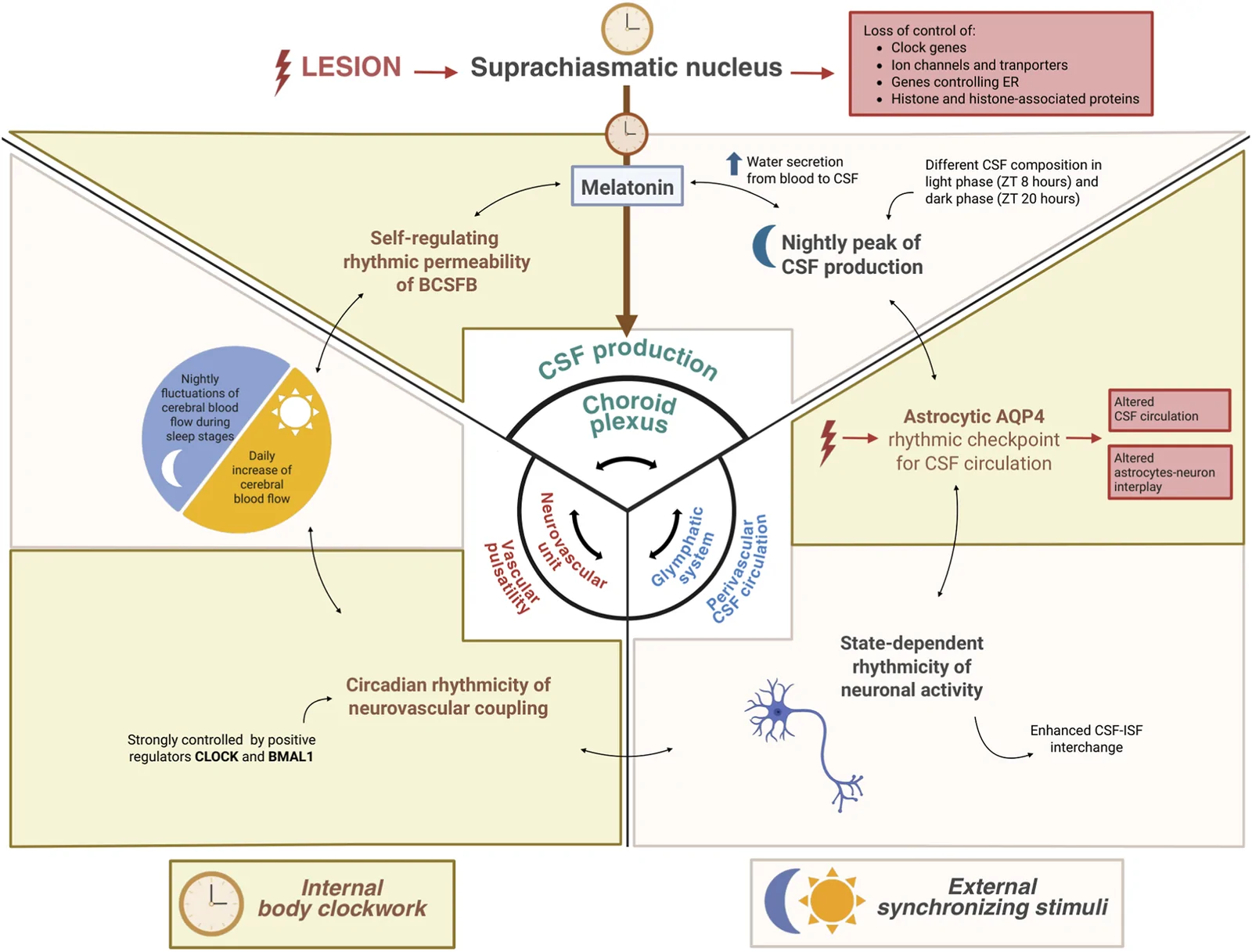
Coordinating influence of internal circadian clock and external synchronizing stimuli on the production, circulation, and distribution of CSF. The figure summarizes the internal circadian clock and external synchronizing stimuli interact in production of CSF and the ability of this system to regulate CSF solutes content and disposal of brain waste product through the neurovascular unit regulation and the glymphatic transport system. External light cues trigger the rest phase in a species-specific manner, promoting state-dependent rhythmicity of neuronal activity that contributes to GS functionality. Notably there is a suprachiasmatic nucleus central clock sensitive to the day/night synchronizing action and regulating the nightly peak of CSF production. The two other compartments, the neurovascular unit and the glymphatic system, in turn are regulated by both independent internal body clockworks and by external synchronizing stimuli. The net activity of the whole system depends upon the orderly functioning of these mechanisms. The importance of regulatory control within each sector is reflected in the relative dimension of the typography. Primary pacemakers and upstream drivers (such as SCN and core clock loops) are indicated by larger fonts, whereas downstream tissue-specific targets, transport channels, and localized physiological responses are identified by increasingly smaller fonts. 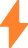
The red arrow highlights disruption and impairment. 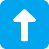 The blue pointing up arrow indicates an increase.

In turn the disruption of the regulation of the above listed clock genes leads to the loss of control of numerous other rhythmic genes including three major subnetworks: clock genes themselves, genes controlling ER protein processing (controlling the ER stress response), and histone associated proteins (Figure 2; Table 1).

**TABLE 1:** Molecular effectors of circadian rhythmicity and impact of day/night alternation on CSF physiology. This table integrates four biological boundaries commonly studied in isolation, the SCN, choroid plexus, glymphatic system, and neurovascular unit, by translating data across humans, mice, rats, ovine and pigs to differentiate conserved mechanisms from rodent-specific results. It provides a dedicated critique of principal limitations, emphasizing a broad dependence on correlative rather than causal evidence in the regulation of circadian and glymphatic processes. Additionally, the synthesized data reveal essential methodological gaps, noting that many findings rely on *in vitro* or *ex vivo* models that lack systemic physiological forces, while human data are constrained by small-sample post-mortem tissue atlases and group-level MRI averages that do not offer individual-level mechanistic detail.

Factor/Gene/System	Clock implication	Proposed functional effect	Species/Experimental model	Type of evidence	Key limitations/Open questions
Suprachiasmatic nucleus (SCN)					
BMAL1	Core clock transcription factor	Controls astrocytic modulation of GABAergic signalling and glutamatergic signalling in SCN neurons	Mouse (Brancaccio et al., 2019; Barca-Mayo et al., 2017)	*In vivo* + *In vitro* (Barca-Mayo et al., 2017); *Ex vivo* SCN slices (Brancaccio et al., 2019)	Evidence from SCN-specific astrocyte manipulations
EAAT3	Rhythmic glutamate transporter	Rhythmic glutamate uptake in SCN; time-of-day dependent activity	Mouse (Brancaccio et al., 2017)	*Ex vivo* (SCN slices)	Demonstrated in SCN *ex vivo* slices
Astrocytic Ca^2+^ oscillations	Glio-neurovascular coupling	Day peak of astrocytic Ca^2+^ in SCN; modulates perivascular fluid dynamics and neurovascular coupling	Mouse (Brancaccio et al., 2019)	*Ex vivo* (SCN slices)	Demonstrated in SCN *ex vivo* slices
DAC (Dystrophin-Associated Complex)	SCN-driven synchronisation of peripheral/regional oscillators via neuronal connectivity and hormonal signalling	SCN-driven synchronisation has been proposed to underlie the phase lags observed in DAC/Aqp4 acrophases across brain regions, interpreted as a physiological consequence of a multi-oscillator architecture	Mouse (Morse et al., 2026)	*Ex vivo*	A causal linking between SCN timing function and circadian regulation of glymphatic clearance is missing
Choroid plexus					
DEX (Dexamethasone)	Per1, Per2 activation	Restores circadian phase; increases amplitude of circadian oscillations	Wistar rats, organotypic explants of the 4th ventricle CP (Liška et al., 2021)	*In vivo* + *In vitro*	The study uses two different rodent species to build its conclusions (wistar rats and *mPer2Luc* mice-for the organotypic explants). The experimental design was based only on the 4th ventricle choroid plexus plus other limitations linked to the non-physiological *in vivo* delivery and *in vitro* treatment conditions
Melatonin	SCN→CP signalling via CSF; paracrine BCSFB effect	Augments water secretion from blood to CSF; regulates claudin-2 expression	Wistar rats, porcine CP explants (Quintela et al., 2018a)	*In vivo* + *Ex vivo*	Different animal species; melatonin is synthesized by porcine CP explants, although without a circadian pattern
AVP (Arginine Vasopressin)	SCN→CP signalling via CSF	Modulates BCSFB permeability and CSF composition	Mouse (hypotalamic brain slices) (Tousson and Meissl, 2004)	*Ex vivo*	Only indirect evidence that SCN signal to CP are through AVP
VIP	SCN→CP signalling via CSF	Modulates BCSFB permeability and CSF composition	Rat (Nilsson et al., 1992)	*In vivo*	Effects observed after VIP intravenous infusion
Serotonin	SCN→CP signalling via CSF	Modulates BCSFB permeability and CSF composition	Monkey (Yan et al., 1993)	*In vivo*	Only indirect evidence from the study of the levels of serotonin in both CSF and plasma
Transthyretin	CP secretory output	Diurnal regulation of transthyretin expression in CP modulates CSF- transthyretin levels	Mouse (Fame et al., 2023)	*In vivo* + *Ex vivo*	There is a sex-specific bias, due to the exclusive use of male mice; for the comprehensive multi-omic mapping only two specific time points (9 a.m. and 9 p.m.) were compared; global Bmal1 knockout mice, displaying, fully disrupted circadian behaviours, were used
NCBE (NBCn2)	CSF ionic composition	Increased expression during dark phase (+11% at ZT20 vs. ZT8); modulates acid-base balance	Human and rodent (Steffensen et al., 2023)	*In vivo*	As indicated also by the authors, there are several key limitations, including the fact that the human CSF sampling was based on patients affected by various clinical acute conditions requiring therapy. Moreover, in the human cohort, the hourly fluid output from an external ventricular drain was used as a proxy for the CSF secretion rate. For the rodent experiments, the authors explicitly acknowledge that the exact absolute quantification of CSF secretion rates in live animals remains challenging. The *ex vivo* radioisotope transport assays (used to evaluate Na^+^/K^+^-ATPase and NKCC1 transporter), were performed on excised choroid plexus isolated from the native cerebrospinal fluid, possibly containing critical neurohumoral signals
WNK1	NKCC1 regulation	Modulates NKCC1 activity; upregulated during dark phase	Human and rodent (Steffensen et al., 2023)	*In vivo*	
NBCn1	CSF ionic composition	Increased expression during dark phase (+21% at ZT20 vs. ZT8); modulates NKCC1 activity	Human and rodent (Steffensen et al., 2023)	*In vivo*	
TRPV4	Osmosensing/water transport at BCSFB	Involved in osmosensing and water transport; modulates NKCC1 via WNK1	Human and rodent (Steffensen et al., 2023)	*In vivo*	
CSF metabolites (Lysine, Arginine, Cytosine, 5-methylcytosine)	CP secretory diurnal variation	Higher levels in light phase (ZT 8) reflect diurnal variation in CP secretory activity	Human and rat (Edelbo et al., 2023)	*In vivo +* *Ex vivo*	In rat experiments, the authors collected samples only at two times (8 a.m. and 8 p.m.), a choice that cannot identify genes expressing complex sinusoidal rhythms; using bulk RNA sequencing of whole choroid plexus tissue is biased by cellular heterogeneity. The human CSF samples were collected at unrecorded times of the day
Glymphatic system					
AQP4	Structural scaffold for clock gene expression	Loss of perivascular AQP4 → loss of rhythmic expression of BMAL1, CLOCK, PER1, PER2, CRY2	Mouse (Yao et al., 2023)	*In vivo* + *In vitro*	Directionality unclear: loss of AQP4 polarization and disrupted clock gene rhythmicity co-occur in CUMS model but causal hierarchy not established; physiological context not tested Per2→Dtna→AQP4 mechanism demonstrated only *in vitro* (primary astrocytes)
DAC (Dystrophin-Associated Complex)	Circadian-regulated DAC components with day/night differential expression	In the brain, the DAC components peak earlier than AQP4, which is most highly expressed during the inactive phase, supporting the hypothesis that the DAC promotes AQP4 polarization and, by extension glymphatic flow	Mouse (Hablitz et al., 2020; Morse et al., 2026); cross-species conservation in chicken and baboon brain/peripheral tissue (Morse et al., 2026)	*In vivo*	Causal hierarchy between DAC rhythmicity and AQP4 polarisation not established
CRY1	Clock gene	Lower CRY1 expression associated with greater myelin volume fraction disparities between good and poor sleepers	Human (MRI + transcriptomic atlas; (Andica et al., 2024))	*In vivo* + *Ex vivo*	Clock gene expression from *postmortem* atlas (6 donors, left hemisphere only), not measured in MRI participants; gene expression correlated with group-averaged regional myelin differences, not with individual-level data. No causal link established
CRY2	Clock gene	Higher CRY2 expression associated with greater myelin volume fraction differences in poor sleepers	Human (MRI + transcriptomic atlas; (Andica et al., 2024))	*In vivo* + *Ex vivo*	
CLOCK	Core clock transcription factor	Higher CLOCK expression associated with greater myelin volume fraction differences in poor sleepers	Human (MRI + transcriptomic atlas; (Andica et al., 2024))	*In vivo* + *Ex vivo*	
Neurovascular unit					
CLOCK	Core clock transcription factor	Loss of CLOCK (ClockΔ19/Δ19) → abolishes circadian oscillation of occludin mRNA and impairs endothelial barrier function	Mouse (reviewed in (Hudson et al., 2019; Schurhoff and Toborek, 2023))	*In vivo* + *In vitro*	Direct causal link between CLOCK-driven transcription and whole-BBB functional rhythmicity remains unclear
BMAL1	Core clock transcription factor	Regulates endothelial and pericyte function via TRPM7 and PDGFRβ signalling; loss → Claudin-5 dysregulation, impaired BBB integrity and age-dependent pericyte loss	Mouse/Rat (Carver et al., 2014; Winkler et al., 2011; Nakazato et al., 2017)	*In vivo* + *In vitro*	Relative contribution of endothelial vs. pericyte BMAL1 signaling to BBB rhythmicity remains unresolved; impact on glymphatic function still indirect
Tight junction proteins (occludin, Claudin-5)	BBB structural integrity	mRNA levels oscillate with circadian rhythm; disrupted in ClockΔ19/Δ19 mice; Claudin-5 absent in mouse CP epithelium (differs from BBB)	Mouse (reviewed in (Hudson et al., 2019; Schurhoff and Toborek, 2023))	*In vivo* + *In vitro*	Functional link between tight junction rhythmicity and BBB permeability/CSF–ISF exchange not established
Pericytes/PDGFRβ	BBB and perivascular tone regulation	BMAL1-dependent PDGFRβ expression regulates pericyte survival and BBB integrity; pericyte loss linked to perivascular dysfunction	Mouse (reviewed in (Winkler et al., 2011; Nakazato et al., 2017))	*In vivo* + *In vitro*	Direct causal link between circadian pericyte regulation and glymphatic flow not experimentally established; contribution to AQP4 polarization and glymphatic function remains indirect
Cerebral blood flow (CBF)	Vascular pulsatility/neurovascular coupling	Decreases at sleep onset (stages I–II), further reduced at NREM III/IV, normalized at REM; nocturnal pulsatility drives perivascular CSF flow	Human/Mouse (Corfield et al., 2006; Iliff et al., 2013)	*In vivo* (human+mouse) + *Ex vivo* (mouse)	Contribution of circadian vs. sleep-state components remains difficult to disentangle; direct quantitative coupling with glymphatic flux still under investigation
ABC transporters (e.g., P-gp)	BBB efflux capacity	Expression and function inversely related to diurnal neuronal activity; lower during wakefulness, higher during sleep to support waste clearance	Mouse (Pulido et al., 2020)	*In vivo* + *Ex vivo*	Mechanistic link between neuronal activity, circadian gene control, and transporter rhythmicity not fully resolved; impact on whole-brain clearance efficiency unclear

The concept that loss of rhythmicity changes gene expression is not new in circadian research, but it may be new applied to the brain fluid compartments dynamics and in particular when considering the interplay between the various components of the systems.

In addition, the dysregulated rhythmic genes include genes regulating WNT signaling and CSF composition as summarized by Sládek et al., (2024). In addition to the former, these authors (Sládek et al., 2024) identified rhythmically expressed genes, which have been associated with developmental processes, associated with hydrocephalus. Examples are genes controlling the movement and accumulation of CSF *Hydin* and Coiled-Coil Domain Containing 88C (*Ccdc88c*) which are associated with developmental abnormalities of the structure of the brain ventricular system (Liu et al., 2024). Notably mutations in these genes cause, through different mechanisms, excessive accumulations of CSF within brain ventricles (Davy and Robinson, 2003; Marguet et al., 2021).

### 3.4 Take-home model of choroid plexus circadian regulation

To synthesize the complex, rhythmic regulation of brain fluid dynamics, we may consider three levels of evidence:

1. Best supported mechanism: nocturnal CSF secretion driven by ion exchangers and modulated by TRPV4/WNK1; the role of AQP1 at the apical level of the CP and of AQP4 at the extrachoroidal/astrocytic level; The hierarchical synchronization exerted by the SCN pacemaker on CP clock genes (*Per1*, *Per2*, *Bmal1*, etc.), as demonstrated by lesion studies.
2. Inferred mechanisms: The action of dexamethasone and endogenous glucocorticoids in reinforcing and resetting the phase of the CP circadian clock both *in vivo* and *in vitro*; the variation in blood-cerebrospinal fluid barrier (BCSFB) permeability through the rhythmic/hourly regulation of the tight junction protein claudin-2.
3. Speculative mechanisms/future research areas: the exact quantitative contribution of AQP4 compared to other extrachoroidal fluid pathways; the signal trafficking model in which the PG acts as a stabilizing “frequency coupler” between the SCN and the CP through melatonin secretion into the CSF.

Within this context, we propose a hierarchical model where the CP acts as a metabolic and fluidic rhythmic hub under both central and local clock control. Accordingly, the SCN drives the overarching 24-h master rhythm. In physiological conditions, in spite of divergent intrinsic oscillatory periods when isolated they are hierarchically synchronized *in vivo* to a strict 24-h cycle, stabilized by the pineal gland via melatonin secretion and direct systemic/neural pathways.

## 4 Glymphatic system

### 4.1 The glymphatic system and the role of AQP4 in brain fluid hydrodynamic

The GS is essential for maintaining the homeostasis of brain parenchyma (Zahran et al., 2026). The perivascular space, the area between the astrocytic endfeet and the smooth muscle and vascular endothelium of the brain vasculature, is thought to be a crucial component for the entire central nervous system (CNS). See also a more detailed description in the paragraph reporting the neurovascular unit and its structure in the various portions of the brain vascular tree.

The GS has been proposed as a brain-wide pathway facilitating interstitial solute clearance through coordinated CSF and ISF movement. In their pioneering study, Needergaard’s group in 2012 (Iliff et al., 2012) demonstrated that CSF enters the parenchyma along para-arterial spaces, mixes with ISF, and exits via paravenous routes, with AQP4-expressing astrocytic endfeet coupling periarterial influx to perivenous efflux, driven primarily by arterial pulsations (Mestre et al., 2018a).

This framework has been enormously influential, stimulating renewed interest in brain fluid dynamics and waste clearance. Its core assumptions, however, have remained the subject of active investigation, and accumulating evidence has led to the partial revision of several original tenets (revised in (Hladky and Barrand, 2022)). Solute transport reflects a combination of convective perivascular flow along preferred routes and diffusion-dominated movement within the parenchymal interstitium (Syková and Nicholson, 2008; Smith et al., 2017). The flow velocity required to sweep solutes toward perivenous spaces would need to exceed CSF production by more than 40-fold, a physiologically untenable condition (Hladky and Barrand, 2022), and only ∼20% of cisternal CSF actually enters the parenchyma under optimised conditions (Lee et al., 2018). ISF secretion across the BBB has been proposed as an independent fluid source to resolve this mass balance deficit, though its rate remains unknown (Hladky and Barrand, 2022). Smith and colleagues in 2017 (Smith et al., 2017) demonstrated using fluorescence recovery after photobleaching that diffusion alone fully accounts for solute movement in undisturbed parenchyma. Furthermore, both influx and efflux occur along periarterial pathways, indicating these spaces are bidirectional rather than exclusively inward-directed (Hladky and Barrand, 2022; Abbott et al., 2018). On current evidence, extravascular solute delivery and removal from the parenchyma are accounted for by the interplay of diffusion, net perivascular fluid flows, convective mixing, and possibly active vasomotion (Hladky and Barrand, 2022).

AQP4 loss or perivascular polarization has been consistently associated with impaired solute transfer (Mestre et al., 2018a; Bojarskaite et al., 2024), although the underlying mechanism is likely indirect rather than a direct consequence of reduced transcellular bulk flow (Hladky and Barrand, 2022). Endfoot gaps appear sufficient for paracellular water movement under physiological hydrostatic conditions, and since AQP4 is impermeable to osmotic active ions, water transport through AQP4 without solute co-movement would generate a local osmotic gradient opposing further flow, suggesting that AQP4-mediated bulk flow may be inherently self-limiting (Smith et al., 2015; Smith and Verkman, 2018). It has therefore been proposed that AQP4 contributes through a more nuanced set of mechanisms: facilitating water movement in response to osmotic gradients generated by solute fluxes, modulating the extracellular microenvironment through interactions with other membrane proteins, and serving a structural role in anchoring astrocytic endfeet at the gliovascular interface (Hladky and Barrand, 2022; Abbott et al., 2018). Upon exposure to altered extracellular and/or intracellular osmolarity, AQP4 activity promotes the efflux or influx of water to balance the movement of ions and osmolytes, resulting in cell shrinkage or swelling. This, in turn, leads to regulatory responses to increase (RVI) or decrease (RVD) cell volume (Franco et al., 2008).

RVD and RVI are homeostatic processes by which astrocytes restore their volume following osmotic perturbations through coordinated ion and osmolyte transport (Lang et al., 1998; Hoffmann et al., 2009; Kimelberg Harold et al., 2006). RVD is triggered by hypotonic swelling and is primarily mediated by the activation of K^+^ and Cl^−^ efflux pathways, leading to osmotically driven water extrusion (Okada, 2004). In astrocytes, this process involves volume-regulated anion channels (VRACs), now identified as heteromers of the leucine-rich repeat containing protein 8 family (LRRC8A–E), which permit the efflux of Cl^−^ and organic osmolytes such as taurine and excitatory amino acids (Murphy et al., 2017; Voss et al., 2014; Lafrenaye and Simard, 2019). To maintain electroneutrality, K^+^ exits the cell through voltage- and Ca^2+^-dependent K^+^ channels, with water following passively (Pasantes-Morales and Vázquez-Juárez, 2012; Kimelberg and O’Connor, 1993). Additional contributors include Ca^2+^-dependent signaling cascades and, in some contexts, Cl^−^ channels such as ClC-2, which is enriched at astrocytic endfeet and functionally associated with AQP4 (Blanz et al., 2007; Benfenati et al., 2007). Conversely, RVI is activated under hypertonic conditions and restores cell volume through net ion uptake, predominantly via Na^+^-coupled transport mechanisms (Wehner et al., 2003). The Na^+^-K^+^-2Cl^-^ cotransporter NKCC1 represents a major component of astrocytic RVI, promoting the electroneutral influx of Na^+^, K^+^, and Cl^−^, thereby increasing intracellular osmolarity and driving water entry (Jayakumar and Norenberg, 2010). In parallel, hypertonicity-induced cation channels (HICCs), including amiloride-sensitive and -insensitive conductances, can mediate rapid Na^+^ influx and contribute substantially to RVI, often exceeding the contribution of Na^+^/H^+^ exchangers (Wehner et al., 2006). Together, RVD and RVI reflect tightly regulated, ion-transport–driven volume control mechanisms that are essential for astrocyte physiology and for maintaining extracellular and perivascular homeostasis in the brain.

As mentioned above, astrocytic Ca^2+^ signals are involved in the mechanisms of cerebral drainage, as they play a key role in detecting a decrease in external osmolarity and in the consequent efflux of water and osmolytes (Pasantes-Morales et al., 2006). Cultured primary astrocytes with AQP4 gene depletion lose the ability to sustain RVD and to increase intracellular calcium levels, which would be a normal response to hypotonic stress (Benfenati et al., 2011). AQP4 is not permeable to the passage of calcium ions, so an osmosensor complex between AQP4 and TRPV4, a polymodal channel that responds to mechanical stress by mediating the influx of Ca^2+^, has been found essential for RVD (Benfenati et al., 2011). The dynamic role of TRPV4 in modulating the Ca^2+^ signal has been further explored at the astrocyte endfoot microdomain level, revealing its contribution to astrocyte-mediated regulation of cerebral microcirculation (Dunn et al., 2013). In particular, the dynamic interaction between TRPV4 and inositol trisphosphate (IP_3_) receptors (IP_3_Rs) was investigated in neurovascular coupling, i.e., the matching between local cerebral blood flow and neuronal metabolism, mediated by astrocytes. Neuronal stimulation caused an increase in IP_3_ levels, leading to an increase in endfoot Ca^2+^ levels and activation of TRPV4 channels. Furthermore, a bidirectional interaction between TRPV4 channels and IP_3_Rs has been observed, where activation of endfoot TRPV4 channels led to Ca^2+^ entry that activated IP_3_Rs, amplifying local Ca^2+^ signals and stimulating nearby IP_3_Rs to produce calcium waves, thus demonstrating a feedback mechanism of Ca^2+^-dependent inhibition of both IP_3_Rs and TRPV4.

Expression and localization of AQP4 can impact the functionality of GS. The perivascular localization of AQP4 can be regulated by the differential expression of its two main isoforms, AQP4-M1 and AQP4-M23, with AQP4-M23 mainly localized to the astrocytic endfeet (De Bellis et al., 2021; Smith et al., 2014). AQP4 polarization is then closely associated with the dystrophin-associated complex (DAC), which includes dystrophin protein 71 (DP71), dystroglycan, and syntrophin, and which performs a scaffold function connecting blood vessels, astrocytes, and AQP4 to stabilize its perivascular localization (Waite et al., 2012). Lack of DP71 perturbs AQP4 aggregation in astrocytic endfeet adjacent to capillaries, resulting in changes in brain water and ion homeostasis as well as vascular permeability (Belmaat et al., 2021), and the ubiquitination-mediated degradation of DP71 disturbs fluid outflow and glymphatic clearance (Yang et al., 2024). Similarly, the cleaved form of the transmembrane beta-subunit of dystroglycan leads to reduced polarization of AQP4 at the astrocyte endfeet (Si et al., 2024). Selective loss of the perivascular AQP4 pool, achieved by alpha-syntrophin knockout which eliminates 90% of perivascular AQP4, impairs the clearance of high but not low molecular weight solutes (Bojarskaite et al., 2024). AQP4 polarity is also regulated by its phosphorylation state (Silberstein et al., 2004): the LRRK2 R1441G mutation leads to accumulation of phospho-AQP4 and reduced glymphatic clearance efficiency in an *in vivo* model of Parkinson’s disease (Huang et al., 2024). Crucially for the scope of this review, DAC components including dystroglycan (*Dag1*), dystrophin (*Dmd*), dystrobrevin alpha (*Dtna*), and alpha-1-syntrophin (*Snta1*) exhibit significant circadian rhythms in gene expression across brain and peripheral tissues, peaking in the late active to early inactive phase (Morse et al., 2026). This rhythmic expression is consistent with the functional output of the glymphatic system, and has been proposed as a key mechanism underlying the time-of-day regulation of AQP4 polarization and waste clearance (Hablitz et al., 2020). Furthermore, PER2 has been shown to negatively correlate with AQP4 polarization by suppressing the expression of α-dystrobrevin, a DAC component that controls AQP4 perivascular anchoring (Yao et al., 2023), thus positioning the molecular clock as a direct upstream regulator of DAC integrity and glymphatic rhythmicity.

Beyond the DAC, several other molecular regulators of AQP4 expression have been documented in the literature, though whether they are subject to circadian modulation remains unexplored and represents an important avenue for future investigation. At the transcriptional level, Nuclear Factor of Activated T cells 5 (NFAT5), also known as TonEBP, has been shown to bind the AQP4 promoter and upregulate its expression under osmotic stress in astrocytes, both *in vivo* (Yi et al., 2013) and in ammonia-exposed primary astrocyte cultures (Rama Rao et al., 2003; Rao et al., 2005). ERK1/2 signaling has been identified as an upstream regulatory pathway for AQP4 across multiple injury models, including scratch injury (Zhang et al., 2019), fluid percussion injury (Rao et al., 2011), manganese exposure (Rama Rao et al., 2010), oxygen-glucose deprivation (Tang et al., 2013), and spinal cord injury (Li et al., 2021). At the epigenetic level, the transcription factor MAFG, acting via interaction with DNA methyltransferase 3 (Wheeler et al., 2020), has been shown to reduce AQP4 protein levels in LPS-stimulated astrocytes undergoing reactive astrogliosis (Cuautle et al., 2024). Whether any of these regulatory axes are gated by the circadian clock, and whether they contribute to the time-of-day variation in AQP4 expression and glymphatic efficiency, remains to be established.

### 4.2 Neuron-astrocyte interplay in glymphatic system activity

Neurons have been found to be important players in coordinating the removal of metabolic waste (Jiang-Xie et al., 2024; Pulido et al., 2020; Holstein-Rønsbo et al., 2023). Neuronal firing in a highly desynchronized manner is at its maximum during the waking state, in order to ensure the execution of highly complex tasks (Harris and Thiele, 2011). Due to desynchronized spiking, the field potentials generated by each neuron cancel each other out, producing only small fluctuations in the movement of the ISF. Experimental evidence from the 2024 work of Jiang-Xie and colleagues (Jiang-Xie et al., 2024) proved this link with chemogenetic, optogenetic, and imaging-based studies demonstrating that suppression of neuronal spiking attenuates CSF–to–ISF perfusion, whereas rhythmic neuronal activity enhances it. Using intracisternal infusion of low–molecular weight fluorescent tracers combined with *ex vivo* imaging, glymphatic influx was shown to be markedly reduced by chemogenetic inhibition of hippocampal neurons, with effects that were more pronounced during sleep than wakefulness, indicating state-dependent coupling between neuronal dynamics and ISF movement. *In vivo* MRI following cisterna magna delivery of gadolinium-based tracers further revealed that local neuronal silencing disrupts parenchymal tracer penetration suggesting a specific role for neuronal activity in regulating ISF transport. Importantly, noninvasive transcranial optogenetic mimicking slow (∼1 Hz) or theta (∼8 Hz) oscillations was sufficient to generate synthetic ionic waves in the interstitial space and significantly potentiate CSF-to-ISF perfusion, as quantified by increased tracer penetration into the hippocampal parenchyma.

During the state of sleep, the brain generates the most rhythmic oscillations: delta (0.5–4 Hz), theta (6–10 Hz), spindle (12–15 Hz) and ripple (140–200 Hz) waves (Buzsáki, 2006), all well-orchestrated and temporally coupled with each other, and participating in brain cleansing (Jiang-Xie et al., 2024). During sleep, indeed, neurons coordinate their action to generate large-amplitude, rhythmic ionic oscillations in the ISF during sleep, facilitating the inflow of fresh CSF through the parenchyma and the removal of metabolic waste products. Using the words of the authors of the work published in 2024 (Jiang-Xie et al., 2024) “neurons that fire together shower together”. Neurons have the ability to not only generate ion waves in the ISF, but also to regulate perivascular flow through neurovascular-coupling (Holstein-Rønsbo et al., 2023), as well as transport across the BBB (Pulido et al., 2020).

Neuronal stimulation has been proposed to negatively influence ISF through two potential mechanisms, biochemical or biomechanical (Shi et al., 2015). In the first mechanism neuronal excitation is followed by a series of chemical activities, such as ion exchange, nutrient consumption, accumulation of metabolic products and release of neurotransmitters (Jin et al., 2013; Branzoli et al., 2013), which can influence the movement of solutes, based on its chemical characteristics. While, in the second mechanism, during neuronal excitation, the consequent cell swelling, mainly on the astrocytes, is accompanied by a reduction in extracellular space volume up to 30% (Nagelhus and Ottersen, 2013), thus squeezing solutes into the extracellular space and blocking hydrodynamic flow (Shi et al., 2015).

In recent decades, the metabolic exchanges between neurons and glia have been suggested as possible mechanism of circadian rhythmicity in mammals (Chi-Castañeda and Ortega, 2017).

A substantial body of evidence demonstrates that astrocytic calcium signaling, morphology, and gene-regulatory functions are dynamically regulated across the entire brain by sleep–wake states and neuromodulatory rhythms (Bojarskaite et al., 2020; Vaidyanathan et al., 2021; Wang et al., 2023). Distinct brain states are characterized by specific patterns of neuronal synchronization, ranging from slow oscillations during non-rapid eye movement (NREM) sleep to desynchronized high-frequency activity during wakefulness, and astrocytes are increasingly recognized as active participants in these global state transitions (reviewed in (Kjaerby et al., 2017)). Noradrenergic and cholinergic systems, which are tightly coupled during arousal and vigilance, converge on astrocytes to induce widespread intracellular Ca^2+^ signaling via Gi- and Gq-coupled receptors, positioning astrocytes as active players of brain-wide neuromodulatory tone rather than passive responders. *In vivo* imaging studies have shown that global astrocytic Ca^2+^ signals emerge seconds after locomotion onset and behavioral arousal, suggesting that glutamatergic and neuromodulatory inputs synergistically promote astrocytic activation during wakefulness (Stéphane et al., 1999; Aoki et al., 1994). Conversely, cortical astrocytic Ca^2+^ signaling is markedly reduced during sleep and exhibits state-specific dynamics, with particularly low activity during intermediate sleep and a pronounced rise preceding transitions from slow-wave sleep to wakefulness (Bojarskaite et al., 2020; Vaidyanathan et al., 2021). Astrocytic GPCR-induced Ca^2+^ has a pivotal role; in particular, Gi-GPCR Ca^2+^ signaling is linked to modulation of slow-wave activity, while Gq- GPCR Ca^2+^ signaling is important for mediating sleep/wake transitions (Vaidyanathan et al., 2021). Genetic disruption of IP_3_ receptor–mediated astrocytic Ca^2+^ signaling leads to altered sleep architecture and impaired slow-wave oscillations, indicating a causal role for astrocytes in stabilizing sleep states and regulating brain rhythms critical for learning and memory (Bojarskaite et al., 2020). Mechanistically, astrocytic Ca^2+^ signaling modulates extracellular neurotransmitter and ion homeostasis, including suppression of GABA uptake, regulation of extracellular K^+^ via Ca^2+^-dependent activation of Na^+^/K^+^-ATPase and cotransporters, and activity-dependent glycogen mobilization to support ionic buffering demands (Wallraff et al., 2006; Seifert et al., 2009; Larsen et al., 2014; Wang et al., 2012; Xu et al., 2013; Choi et al., 2012). In parallel, noradrenergic signaling induces rapid astrocytic morphological remodeling through β-adrenergic and cAMP-dependent pathways, further linking astrocyte structure and metabolism to global brain state transitions (Prebil et al., 2011; Kreft et al., 2012). The locus coeruleus and its neurotransmitter norepinephrine play a major role in modulating these behaviors by switching between small Ca^2+^ signaling during sleep to greater Ca^2+^ signaling in the arousal state, enhancing sensory input and responsiveness during arousal and inhibiting sensory input during sleep (Wang et al., 2023). Together, these findings indicate that astrocytic Ca^2+^ signaling and associated structural and metabolic adaptations represent a brain-wide mechanism through which sleep–wake and neuromodulatory rhythms shape neuronal network function.

### 4.3 Physiological regulation of glymphatic system and circadian activity

The regulation of the GS reflects a sophisticated interplay between state-dependent sleep-wake cycles and endogenous circadian rhythms. While often studied together, these processes represent distinct regulatory scales. Sleep research typically focuses on high-frequency, state-dependent oscillations, such as slow-wave activity and spindles, which induce rapid, high-magnitude changes (up to 200%) in perivascular fluid movement during specific sleep bouts (Xie et al., 2013; Hablitz et al., 2019). Conversely, circadian research characterizes endogenous, low-frequency oscillations that persist over multiple days, months, or even years, regardless of immediate arousal states (Hablitz et al., 2020). Understanding how these two systems are both separate and intertwined is essential for characterizing brain homeostasis and metabolic waste clearance (Miyakoshi et al., 2023; Deboer, 2018).

Specific sleep architectures are critical for GS functionality. Sleep disturbances, such as fragmentation, absence of NREM III, and shorter total sleep duration, impair glymphatic function (Xie et al., 2013; Ulv Larsen et al., 2025; Vasciaveo et al., 2023), thus decreasing the removal of metabolic waste, as evidenced by human studies showing β-amyloid accumulation after even a single night of sleep deprivation (Shokri-Kojori et al., 2018; Fultz et al., 2019). These state-dependent changes are dramatic (Miyakoshi et al., 2023; Hauglund et al., 2025; Miao et al., 2024). According to a two-photon imaging experiment, mice under anesthesia or natural sleep had a 60% larger interstitial space and cleared waste products, such as beta-amyloid peptide, twice as quickly as awake mice (Xie et al., 2013).

Independent of the sleep state, the GS exhibits endogenous circadian rhythmicity, with peak clearance efficiency typically observed during the rest phase (e.g., the light phase in nocturnal rodents) (Hablitz et al., 2020; C et al., 2020). This regulation is mediated by the rhythmic polarization of AQP4 at the astrocytic endfeet. Indeed, the polarization and gating of AQP4 are modulated by specific kinases, such as protein kinase C and calcium/calmodulin-dependent protein kinase II whose activity is under direct control of the molecular clock (Kitchen et al., 2020). These circadian-driven post-translational modifications ensure that AQP4 membrane efficiency aligns with the 24-h metabolic demands of the brain, demonstrating that GS regulation involves a complex interplay between the circadian system and the sleep-wake cycle rather than being driven by either exclusively (Mestre et al., 2018b).

Recent findings (Morse et al., 2026) confirm that this circadian rhythmicity extends to the DAC itself, as introduced above: AQP4 and DAC expression consistently peak in the late active to early inactive phase, consistent with the increased CSF influx and solute clearance from the brain observed in the inactive phase (Hablitz et al., 2020).

AQP4 absence has been reported to exacerbate the prolonged light-induced impairment of circadian oscillations in AQP4-knockout mice (Murakami et al., 2023). Moreover, the circadian regularity in glymphatic fluid transport is largely eliminated when AQP4 is deleted. The disruption of the astrocytic clock has been linked to impaired glymphatic clearance and neurovascular dysfunction, primarily through the regulation of AQP4 polarization (Musiek et al., 2013; Lananna et al., 2018). In particular, the circadian rhythm regulates the high polarization of AQP4 in astrocytic endfeet, in turn affecting solute clearance, CSF–ISF exchange, and bulk fluid flow (Hablitz et al., 2020), thus indicating that astrocytes and AQP4 serve as a finely regulated rhythmic checkpoint for the functioning of the GS. The pivotal role of the astrocytic molecular clock, specifically BMAL1, in the hierarchical coordination of neuronal oscillators is well established, with its essential role in maintaining AQP4 distribution and preventing neuroinflammation (Lananna et al., 2018; Barca-Mayo et al., 2017), and recent evidence links the polarized expression of AQP4 in perivascular astrocytes to the loss of rhythmic expression of core clock proteins (CLOCK, BMAL1, PER1, PER2, and CRY2), consistent with the PER2-dependent regulation of α-dystrobrevin described above (Yao et al., 2023).

However, given the known functional redundancy between Period isoforms (*Per1*, *Per2*, and *Per3*), the observed correlation does not necessarily imply an exclusive regulatory role for *Per2*. It is possible that compensatory mechanisms among these isoforms maintain some degree of astrocytic rhythmicity, a factor that warrants further investigation using isoform-specific knockout models.

Moreover, the astrocyte clock has been hypothesized to influence, if not cause, the circadian differences in polarization of AQP4 thus influencing both glymphatic influx and drainage to the lymphnodes (Hablitz et al., 2020). Astrocyte clock dysfunction may result in curtailed or abnormal sleep, with the consequent impact on the glymphatic function. Selective genetic manipulation of mouse SCN astrocytes, with specific *Bmal1* deletion, resulted in a loss of their circadian rhythmicity, thus suggesting their important role in regulating the free-running periodicity of the mammalian master clock (Tso et al., 2017).

Interestingly, a potential relationship among GS misalignment, poor sleep quality, and myelin integrity alterations, with relative impact on circadian clock gene expression has been reported (Andica et al., 2024). Poor sleep quality was found to be related to lower brain myelin integrity, evaluated by means of myelin volume fraction. This association may be mechanistically driven by the impairment of glymphatic-mediated clearance during sleep deprivation; the resulting accumulation of metabolic waste and pro-inflammatory mediators in the interstitial space can trigger oxidative stress and oligodendrocyte dysfunction, thereby compromising myelin maintenance and repair (Hsu et al., 2023). In addition, brain regions with higher expression of CLOCK, Cryptochrome Circadian Regulator 2 (CRY2), PER1, and PER2 showed higher myelin volume fraction disparities when comparing good and poor sleepers, whereas lower expression of Cryptochrome Circadian Regulator 1 (CRY1) was associated with more pronounced differences.

## 5 Neurovascular unit

### 5.1 Role in CSF hydrodynamic and composition

The NVU has attracted interest in recent years due to its central role in maintaining brain homeostasis by coordinating cerebral blood flow (CBF), BBB integrity, and metabolic waste removal. Indeed, beyond its classical function (e.g., providing nutrients and vascular regulation), the NVU is now recognized as a fundamental modulator of CSF dynamics and GS activity, particularly in a circadian-dependent manner (Iadecola, 2017).

The NVU was first defined as a structural and functional unit comprising endothelial cells, vascular smooth muscle cells (VSMCs), pericytes and astrocytic end-feet (Kaplan et al., 2020). More recently, growing evidence indicates that the NVU is not a uniform entity throughout the cerebral vasculature, but rather a heterogeneous system depending on the position along the vascular tree and by region-specific functions (Schaeffer and Iadecola, 2021).

This heterogeneity is both morphological and transcriptomic, resulting in functionally distinct NVUs across different vascular segments. Large cerebral arteries are characterized by multiple layers of VSMCs surrounding the endothelium, whereas smaller arterioles display a progressive reduction in VSMC coverage and distinct perivascular arrangements (Hannocks et al., 2018). In capillaries, VSMCs are replaced by pericytes or direct astrocytic contacts, while veins exhibit thinner, less contractile VSMC layers associated with perivascular spaces. In parallel, endothelial cells display marked transcriptomic diversity depending on vessel type and brain region, with differential expression profiles related to structural integrity, transport, metabolism, oxygen sensitivity and inflammatory responses (Vanlandewijck et al., 2018; Saunders et al., 2018).

Overall, the impossibility of finding a single NVU model reflects not only the different specialization of brain areas but also the adaptation to changes in systemic and brain factors. Among systemic factors regulating CBF, one of the most important is represented by arterial pressure whose fluctuations are largely regulated by VSMCs through the modulation of Ca^2+^ fluxes (Kawano, , 2011). In addition, CBF is highly sensitive to arterial blood gas composition. Hypercapnia and hypoxia both induce CBF increase due to VSMC relaxation, mediated by changes in CO_2_, pH and nitric oxide (NO) signalling (Hoiland et al., 2019).

Among the factors able to influence CBF a pivotal role is surely represented by sleep. Indeed, it is well-known that during sleep the brain performs the clearance of waste metabolites essential to maintain cerebral homeostasis. In particular, sleep onset (stages I-II), corresponds to a decrease of arterial pressure and an increase of CBF. At stages III/IV (corresponding to non-rapid eye movements sleep), a reduction of CBF and brain metabolism has been observed. At rapid eye movements (REM) sleep stage, CBF and brain activity are normal and finally, at awakening stage, despite the increase in arterial pressure, a CBF reduction has been recorded (Corfield et al., 2006).

These CBF fluctuations are associated with the glymphatic function, consisting in exporting the interstitial fluid to the CSF and to brain vessels and lymphatic system, and could explain why GS is more effective during sleep. Moreover, the restorative effect of sleep may be also explained as the enhanced removal of waste products accumulated during wakefulness in CNS (Xie et al., 2013). On the contrary, the impaired arterial pulsatility observed in ageing brains, may be associated with accumulation and deposition of neurotoxic proteins, including amyloid β (Iliff et al., 2013). The movement of CSF through perivascular spaces leads to the clearance of metabolic waste in the brain and this flow was assured by the expression and correct localization of AQP4 on the vascular endfeet of astrocytes (Iliff et al., 2012).

The alteration of glymphatic function during sleep has been associated to the alteration of AQP4 polarization on astrocytes following the circadian rhythm which in turn is a consequence of the rhythmic gene expression of DAC fundamental components such as dystroglycan 1 (*Dag1*), dystrophin (*Dmd*), dystrobrevin alpha (*Dtna*), alpha-1-syntrophin (*Snta1*) (Hablitz et al., 2020).

Circadian timing has not only a key influence on astrocytes proteins distribution (i.e., AQP4) but has also a deep impact on other components of the NVU of the BBB. In particular, the expression of functional proteins in endothelial cells and pericytes are strongly influenced by circadian rhythm oscillations. First of all, the tight junction proteins, fundamental for maintaining the integrity of the endothelial layer and its barrier effect, exhibit modulation of mRNA and protein levels, depending on circadian rhythm. In particular, occludin mRNA follows circadian rhythm oscillations and loses its time-dependence in circadian altered mice (Clock^Δ19/Δ19^) leading to the loss of endothelium barrier function. Claudin-5 dysregulation is influenced by the circadian clock gene *BMAL1* knockdown, in the inner retina, thus compromising the barrier function (Schurhoff and Toborek, 2023; Hudson et al., 2019). ATP-binding cassette (ABC) efflux transporters represent another target influenced by circadian rhythm. Indeed, the ABC efflux transporters’ expression and function in BBB seems to be inversely related to diurnal neuronal activity. This behavior can be justified by the fact that a lower expression and function in the endothelial cell during daily activity allows the requested neurotransmission, while higher expression and function are useful to the waste clearance process during sleep (Pulido et al., 2020; Cuddapah et al., 2014). Circadian rhythm could also have an impact on BBB function by altering the magnesium transporter (TRPM7) function. Indeed, the circadian clock gene *BMAL1* binds the promoter of TRPM7, influencing its expression and, in turn, the levels of magnesium that represents a fundamental cofactor for the efflux (Zhang et al., 2021). An interesting experiment has been performed by Carver and co-workers, on astrocytes and endothelial cells, to investigate the circadian rhythm-associated expression of circadian clock genes and enzymes. The results showed that the expression of circadian clock genes and enzymes involved in the conversion of arachidonic acid to epoxyeicosatrienoic acids (such as CYP450 epoxygenases, Cyp4x1 and Cyp2c11), was higher during the early diurnal hours (starting at 6 a.m. to 12 p.m.) and the formation of epoxyeicosatrienoic acids exhibited a significant 12 h period rhythm. On this basis, the authors speculated that this rhythmic production of epoxyeicosatrienoic acids, may contribute to circadian changes in blood flow and influence the risk of cardiovascular injuries throughout the day (Carver et al., 2014).

Regarding pericytes, their recruitment and function in the basement membrane of microvessels seem to be influenced by the circadian clock gene *BMAL1*. In particular, platelet-derived growth factor receptor β (PDGFRβ) plays a fundamental role in pericyte recruitment and action because PDGFRβ signaling in pericytes promotes the maintenance of BBB integrity by these cells (Winkler et al., 2011).

Knockdown of *BMAL1* downregulated PDGFRβ transcription in pericytes of brain blood vessels and BMAL1 deletion induced age-dependent loss of pericytes contributing to BBB hyperpermeability. According to these findings the circadian function is related to BBB integrity by the BMAL1-dependent regulation of pericytes, and pericyte-mediated regulation of capillary tone and blood–brain barrier integrity may also influence glymphatic activity. Indeed, pericytes contribute to vascular pulsatility and cerebral blood flow, which drive perivascular fluid movement, and their dysfunction has been linked to altered astrocytic AQP4 polarization, a key factor for efficient glymphatic transport (Nakazato et al., 2017).

### 5.2 The NVU contribution to the waste clearance

The waste clearance in the brain is carried out by the GS, but the NVU has a fundamental influence on the GS. Indeed, previously their roles have been considered a sum of two distinct functions: the cerebrovascular function that assured an adequate CBF, able to supply nutrients, oxygen and so on, and the glymphatic function dedicated to the waste clearance, through the flow of interstitial fluid (Holstein-Rønsbo et al., 2023; Mestre et al., 2020). Currently, the contribution of the NVU to the GS, although under study, seems to overcome this sum of separated functions and delineate a model in which the NVU influences the glymphatic function. Indeed, the flow of CSF in the perivascular spaces (both the periarterial and the perivenular ones) is surely influenced by CSF production force but also by the arterial pulsations (Kedarasetti et al., 2020). In particular, the heartbeats are transferred to the cerebral arteries as pulsations and these pulsations are, in turn, transferred to periarterial spaces, influencing the driving force by which CSF flows in the GS (Mestre et al., 2018a; Iliff et al., 2013). This synchronization between vascular circulation and glymphatic function has been observed under physiological conditions, but has also been confirmed through pre-clinical experimental models. Indeed, experiments carried out on retired breeder rats subjected to multiple microinfarctions to induce vascular dementia, have demonstrated that these rats presented axonal damage with white matter lesions but, at the same time, exhibited significantly dilated perivascular spaces and decreased expression of AQP4 in the vascular endfeet of astrocytes. These alterations resulted in delayed CSF flow into the brain parenchyma, and reduced waste clearance, leading to glymphatic dysfunction. This study has been conducted on animals with no previous vascular diseases, so the vascular injury represented by the multiple microinfarctions, used to induce dementia, could be considered the initiating event which led to the observed glymphatic dysfunction (Venkat et al., 2017). In patients suffering from alpha-synucleinopathies, the progression towards dementia or motor deterioration is accelerated by both sleep and nocturnal blood pressure alterations and these findings, although they do not directly demonstrate an interconnection between the NVU and the GS, described NVU relevant mechanisms that may influence processes relevant to glymphatic transport (Fagiani et al., 2022). These findings seem to suggest that the alteration of the physiological nocturnal decrease of blood pressure, coupled with the lack of specific sleep stages, could lead to the impossibility of waste clearance normally operated by GS during sleep and in low blood pressure conditions. Indeed, experimental studies demonstrated that interstitial space was found to be significantly expanded during sleep and vascular low-pressure condition, and this event allows an increase of interstitial fluid flow and a consequent removal of misfolded proteins (Xie et al., 2013; Buongiorno et al., 2023). Although the exact description of the interconnection between NVU and GS still needs to be further delineated, their functions could be described as a single *continuum* necessary to maintain neuronal homeostasis and ensure functioning even in the presence of the high metabolism of the brain.

## 6 Limitations of current evidence and open challenges

A number of conceptual and methodological constraints limit the current state of the literature on the circadian orchestration of neurofluid dynamics. Nocturnal rodent models (*Mus musculus* and *Rattus norvegicus*) provide the great bulk of mechanistic information about clock gene regulation of AQP4 polarization and CP transporter dynamics. Accurately matching circadian phases is still a major experimental challenge because of inverted sleep-wake patterns compared to diurnal humans.

Moreover, it is difficult to separate the physiological impacts of sleep states, varying metabolic demands, postural variations, and the suppressive effects of anesthetics on neurovascular coupling from the independent contributions of the endogenous molecular circadian clock. Instead of capturing a pure circadian readout, several experimental methods capture a mixed state.

Technical and ethical limitations prevent direct, real-time measurement of glymphatic flux in people. The majority of current surrogates rely on intrathecal gadolinium injections monitored by MRI, which yields macroscopic clearance profiles but lacks the cellular resolution necessary to conclusively link human waste clearance to particular molecular clock configurations or local AQP4 endfeet density.

By expanding conventional observations to the NVU, this review is an intentional attempt to build a comprehensive framework of circadian neurofluid dynamics. However, a major limitation is that, in order to increase our existing understanding, we are making connections between previously separate parts of the literature. There are still few or highly compartmentalized detailed investigations that analyze the precise circadian molecular clocks governing important key players, such as localized endothelial/pericyte transporters and the upstream regulators of AQP4 polarization. As a result, some components of our integrated model act as a theoretical link to direct mechanistic validation in the future (summarized in Table 1).

The absence of sex- and gender-dependent effects is another significant drawback of this review that represents a systematic blind area in the current state of neurofluid research. Glymphatic clearance and CSF dynamics are believed to be influenced by hormonal changes, differences in neurovascular compliance, and sexually dimorphic clock-gene expression profiles. Unfortunately, we are unable to reach firm, sex-specific results in this general model since fluid dynamics data are not stratified by biological sex in the present main literature. Similar observations apply to the effect of drugs, which may specifically affect circadian rhythmicity of various biological clocks also at the level of brain fluid dynamic control, but have not been systematically investigated.

## 7 Conclusion

The peculiar crosstalk between the intrinsic biological clock and the described signaling pathways highlights the importance to consider the temporal variable to better understand the physiopathology of brain fluid circulation and its involvement in the etiopathogenesis of neuropsychiatric diseases. This knowledge will also help to improve preventive and therapeutic management of these diseases. In particular, deciphering these cell-specific circadian systems will be essential for both comprehending fundamental brain physiology and creating chronotherapeutic approaches to alleviate neurodegenerative disorders that are marked by metabolic waste accumulation and glymphatic failure.

We propose an integrated conceptual model for synchronization and primary regulatory mechanisms governing brain fluid dynamics in the brain, addressing on how biological clocks, present at various levels of the finely tuned CSF/interstitial fluid/glymphatic exchange chain, orchestrate these activities and their relationship with the NVU at the neuron/astrocyte/endothelium interface. The circadian cycling of the glymphatic waste removal system is widely recognized; however, as underscored in the review, the precise mechanisms governing the inter-segmental coordination and the anatomical localization of the principal oscillators are still under active debate and a better comprehension is needed.

The review also underscores the need to consider the various portions of the brain fluid circulation (CBF, CSF production and flow, glymphatic system, NVU) as a part of a highly interdependent and regulated system. The crucial need to elucidate the multi-level regulation of specific clock genes is becoming increasingly apparent, as is the question of whether their compensatory function can be assumed by other core circadian clockwork genes.

## Glossary

CSF: Cerebrospinal fluid
CP: Choroid plexuses
ISF: Interstitial fluid
GS: Glymphatic system
BCSFB: Blood-cerebrospinal fluid barrier
AQP1: Aquaporin-1
AQP4: Aquaporin-4
SCN: Suprachiasmatic nucleus
PG: Pineal gland
AVP: Arginine vasopressin
VIP: Vasoactive intestinal polypeptide
DEX: Dexamethasone
*Per1*: Period circadian protein homolog 1
*Per2*: Period Circadian Regulator 2
NCBE: Na+-driven Cl-/HCO3- exchanger
NBCn1: Sodium bicarbonate cotransporter 3
NKCC1: Na–K–Cl cotransporter 1
TRPV4: Transient receptor potential cation channel subfamily V member 4
WNK1: Lysine deficient protein kinase 1
*nr1d1*: Nuclear Receptor Subfamily 1 Group D Member 1
*Ciart*: Circadian Associated Repressor Of Transcription
*Chrono*: ChIP-derived repressor of network oscillator
*Bmal1*: Basic helix-loop-helix ARNT-like protein 1
*Ccdc88c*: Coiled-Coil Domain Containing 88C
CNS: Central nervous system
BBB: Blood-brain barrier
RVI: Regulatory Volume Increase
RVD: Regulatory Volume Decrease
**IP**_**3**_: Inositol trisphosphate
**IP**_**3**_ **Rs**: Inositol trisphosphate receptors
NFAT5: Nuclear factor of activated T cells 5
TonEBP: Tonicity-responsive enhancer binding protein
ERK: Extracellular regulated protein kinase
LPS: Lipopolysaccharide
DAC: Dystrophin-associated complex
DP71: Dystrophin protein 71
LRRK2: Leucine-rich repeat kinase 2
EAAT3: Excitatory amino acid transporter 3
CLOCK: Circadian Locomotor Output Cycles Kaput
CRY2: Cryptochrome Circadian Regulator 2
CRY1: Cryptochrome Circadian Regulator 1
NVU: Neurovascular unit
VSMCs: Vascular smooth muscle cells
CBF: Cerebral blood flow
NO: Nitric oxide
REM: Rapid eye movements
*Dag1*: Dystroglycan 1
*Dmd*: Dystrophin
*Dtna*: Dystrobrevin alpha
*Snta1*: Alpha-1-syntrophin
ABC: ATP-binding cassette
TRPM7: Magnesium transporter
PDGFRβ: Platelet-derived growth factor receptor β
AQP5: Aquaporin-5
AQP7: Aquaporin-7
**Na** ^**+**^ **/K** ^**+**^ **ATPase**: Sodium–potassium pump
NBCe2: Electrogenic sodium bicarbonate cotransporter 4
AE2: Solute carrier family 4 member 2
KCC1: Potassium-chloride transporter, member 4
KCC4: Solute carrier family 12 member 7
Kir7.1: Potassium inwardly-rectifying channel, subfamily J, member 13
MCT4: Monocarboxylate transporter 4
MCT1: Monocarboxylate transporter 1
MCT2: Monocarboxylate transporter 2
MCT3: Monocarboxylate transporter 3
NHE-1: Sodium-hydrogen antiporter 1
